# Malpractice in Radiology: What Should You Worry About?

**DOI:** 10.1155/2013/219259

**Published:** 2013-04-03

**Authors:** Alessandro Cannavale, Mariangela Santoni, Paola Mancarella, Roberto Passariello, Paolo Arbarello

**Affiliations:** ^1^Department of Radiological Sciences, “Sapienza” University of Rome, 324 Viale Regina Elena, 00161 Rome, Italy; ^2^Department of Anatomical, Histological, Forensic Medicine and Orthopedic Sciences, “Sapienza” University of Rome, 336 Viale Regina Elena, 00161 Rome, Italy

## Abstract

Over recent years the professional role of the radiologist has been evolved due to the increasing involvement in the clinical management of the patient. Radiologists have thus been increasingly charged by new duties and liabilities, exposing them to higher risks of legal claims made against them. Malpractice lawsuits in radiology are commonly related to inappropriate medical care or to the poor physician-patient relationship. In the present paper, we provide overview of the basic principles of the medical malpractice law and the main legal issues and causes of legal actions against diagnostic and interventional radiologists. We also address some issues to help radiologists to reduce risks and consequences of malpractice lawsuits. These include (1) following the standard of care to the best of their ability, (2) cautious use of off-label devices, (3) better communication skills among healthcare workers and with the patient, and (4) ensuring being covered by adequate malpractice insurance. Lastly, we described definitions of some medicolegal terms and concepts that are thought to be useful for radiologists to know.

## 1. Introduction

Over the last years radiologists have become substantial part of the clinical-therapeutic management of patients. This implies new liabilities and duties related to interventional procedures which are now added to already existing professional liability from diagnostic exams. Errors in radiology practice are quite common amounting for about 4% of radiologic interpretations rendered by radiologists [1]. Hence, most of the committed errors are of such minor degree or are resolved before a patient's injury may happen.

Nevertheless many errors in clinical practice may harm the patients leading to medical malpractice lawsuits [1].

A lot of the problems of medical malpractice generally are related to two issues: the physician-patient relationship or improper medical care leading bodily harm.

Both knowledge of state law and appropriateness criteria may help the physician to prevent complications and thereby any legal issues with patients.

In the first part of our review we overviewed the current laws and rules concerning the medical malpractice among different countries. 

Moreover we reported frequency and burden of causes of malpractice among radiologists and the related legal sequelae. 

Finally four pieces of advice to reduce the risk to incurring a malpractice complaint are outlined: (1) follow as long as possible the standard of care; (2) be careful in the off-label use of devices (3); improve communication skills with colleagues, nurses, technicians, and, overall, patients, (4) remember the insurance. 

## 2. Overview of Malpractice in Radiology

The malpractice phenomenon in radiology is differently represented worldwide. 

A recent US nationwide research on malpractice suits [2] showed that the most common cause of medical malpractice suits against radiologists was error in diagnosis (mainly failure to diagnosis instead of delay); the category next in frequency was procedural complications, followed by inadequate communication with either patient or referring physician. The most common complication in radiologic exams is the vascular injury (during angiography and other interventional procedures) accounting for 1.31 (95% CI: 1.06, 1.63) claims per 1000 person-years, which can become the subject of a lawsuit.

Conclusion of this study showed that errors of diagnosis (14.83 claims per 1000 person-years) are the most common generic cause of malpractice suits against radiologists. 

Imaging findings related to the breast (4.13 claims per 1000 person-years) were the most common cause of organ-related misdiagnosis subject to malpractice suits. 

Errors of communication with either referring physicians (0.71 claim per 1000 person-years) or patients (0.40 claim per 1000 person-years) are an infrequent cause of malpractice suits against radiologists. 

Failure to recommend additional imaging studies (0.41 claim per 1000 person-years) is an even less common reason for the initiation of lawsuits against radiologists.

In Italy and other European countries there has been reported a frequency of 44 per 1000 cases to be sued with a yearly incidence ranging between 3.6 to 12.6%. Fileni and Magnavita [3] analyzed the insurance claims of Italian radiologists from 1993 to 2006. They found 11.5% of claims for radiologic exams (164 cases), most of which (98 cases) involved interventional radiologic procedures.

In this analysis the main cause of lawsuit was the misdiagnosis (of fracture, cancer, or others) accounting for 66.7% of all causes. Errors in radiological techniques and procedures (contrast, enema, and intervention) awarded the second place accounting for 10.3% of claims (112 cases), almost half of which (59 cases) involved interventional radiological techniques. The most common organ and site of diagnostic errors was the skeletal system (44.5%), followed by the breast (25.8%), the chest (11.4%), and the abdomen (8.3%).

Regarding interventional radiology, Magnavita et al. [4] reported a risk to be sued of 47.3 per 1,000 procedures, meaning that there is a statistical certainty of being sued on average every 21.1 working years.

Leading causes of malpractice claims in interventional procedures were vascular injuries (43.9% of cases) and complications after needle biopsies (14.3%) or after scleroembolisation or thermal ablation (12.2%). Only in 3-4% of cases claims occurred for spinal lesions, failure to perform or delay in performing an investigation or failure to obtain informed consent or provide inadequate information to patients or their relatives.

In England a retrospective research from 1995 to 2006 similarly showed that the largest number of claims (199) concerned delayed or missed diagnoses of cancer, and 73 of these were related to breast radiology [5]. The second most common cause to be sued is missing diagnosis in skeletal radiology amounting to 124 claims.

A Dutch study [6] outlined that an interprenation of screening mammography represents one of the most difficult tasks in radiology and that there are high public's expectations of the efficacy of this imaging technique, thereby missing breast cancer and delaying the diagnosis may have major legal consequences for the screening radiologist.

Hence they conclude that a substantial proportion of screen detected cancers and interval cancers have been missed at a previous screen, and 5% of women experienced a delay in breast cancer diagnosis after referral.

A survey analysis in 1503 German radiologists [7] showed that lawsuits involved the assessment of examinations (38%) and the actual performance of an examination (30%) but not providing information to patients. Radiography (20.2%), angiography (18.4%), and mammography (16.4%) were the most frequent imaging techniques which underwent law proceedings that led to civil (30% of cases) or criminal convictions (5.5% of cases).

Considering both US and European studies accounting cases of malpractice in radiology, an increasing trend of risk to be sued is notable. Both in US, and in European countries the major risk to be sued for radiologists seems to be related to error in diagnosis particularly in breast and skeletal radiology.

Procedural complications related to interventional procedures represent the second most frequent cause of claim made.

Although causes of a malpractice action are similar around the world, the risk of legal complications in Italy is progressively approaching that of the United States where it is estimated that 40% of radiologists are taken to court on average once every 5 years [3].

Many rules and laws across different countries have a substantial influence on the practice of radiology and interventional radiology. 

For example in the USA, medical malpractice is generally under the authority of the states, not under the federal government. Differently from other countries, the framework and legal rules governing malpractice actions are largely established through decisions in lawsuits in state courts, varying among different states/regions in contrast with many other countries [2].

The law that developed concerning medical malpractice is a part of the more general body of law dealing with injuries to people or property, called “tort law.” The law of torts is derived from a combination of common-law principles and legislative enactments. English common law refers to the legal system of England and Wales and forms the basis of jurisprudence in the United States and in many other Commonwealth countries [5].

Hence jury trials are less common in UK, but the legal handling of malpractice claims is otherwise similar to the United States. The National Health System Litigation Authority (NHSLA) coordinates and collates claims against health professionals in the English NHS.

Differently from the common law system, in Italy and other European countries malpractice workup is sustained by civil and penal regulations, not by a “tort law.” According to the Italian Penal and Civil Code medical malpractice is based on “… negligence, imprudence and unskilfulness or failure to comply with laws, regulations, orders, and disciplines.” Physicians in case of negligent conduct are liable to both persecution in criminal court and civil action. Crimes for negligent personal injury are suitable for prosecution by the patient [8].

In Germany, medical malpractice claims are initially referred to mediation boards made up by experts designated by Germany Physicians' Guild. In terms of establishing liability, the German courts adopt a stricter approach. Hence judges, but not juries, try German civil cases and standardized reimbursement tables for noneconomic losses to guide judges' decisions [9].

Whereas the application of the Bolam/Bolitho standard in English courts has largely left what constitutes acceptable medical practice to expert opinion [10], German courts have been more critical in their analysis of the value and credibility of expert opinion. Sweden, Finland, Denmark, and Norway also operate out-of-court, no-fault systems for medical malpractice, designed to compensate patients for negligence they suffer from avoidable risk and complications related to medical care [9]. The systems also compensate patients for injury caused by defective equipment, the misuse of equipment, incorrect diagnoses, and infection contracted during treatment.

Generally medical malpractice has been strictly related to the main tort that is the “negligence.” However the two concepts are not the same: medical malpractice is an active disregard for the necessary steps to provide accurate and ethical health assistance. Medical negligence is a breach of duty or a failure to comply with certain standards [1]. Negligence is often associated with inattention on the health care provider's part and can result from poor doctor-patient communication [11].

In general negligence can be justified when all of the following issues exist: (1) the physician was duty bound to take care, (2) he was in breach of that duty, that he was careless, (3) the patients was injured in direct result, and (4) there was a causal relationship between the injury and the alleged breach of care [12].

The recent increase of malpractice suits is partially attributed to the surge of people seeking medical assistance, combined with the lack of sleep experienced by many hospital professionals [9].

 Once a person brings a malpractice lawsuit, the person (called the “plaintiff”) must show that they were actually under the care of the physician (or other provider) they are suing. The concept is that physicians (or other providers) owe a duty to their patients to use reasonable care and diligence in their treatment.

In merit of this, another landmark of a law suit is the demonstration of “the standard of care” applied or less by the physician. The definition of the standard of care may vary from jurisdiction to jurisdiction. A legal definition of standard of care is “the watchfulness, attention, caution and prudence that a reasonable person in the circumstances would exercise; failure to meet the standard of care is called negligence.” 

The next step is to prove if the physician is shown to have provided substandard care. If he did so, the plaintiff still must prove that the substandard care caused their injury.

In addition to the standard of care, the choice of expert witnesses is crucial and will often determine the outcome of the lawsuit.

The expert witness should have clinical and teaching experience in the type of radiological exam/intervention subject to the malpractice claim and also have experience in testifying in medical malpractice lawsuits. His role is paramount because he replaces the judge's lack of knowledge in specific issues related to medicine and radiology, so that he can be called “the bridge” between the law and the medicine.

Last but not least problem with a lawsuit is the amount of spent resources in terms of time and money.

However the main issue of a malpractice lawsuit seems to be highlighted in 1977 by Berlin who raised the following question: “Does the “missed” radiographic diagnosis constitute malpractice?” [13] After the first tort reform in USA, such question could sound as “passed matter,” however it should be considered still valid due to ambiguous effects of tort reform on medical practice. Nevertheless due to its variability and often less clarity the tort reform issue is highly debated in USA, so that a second tort reform seems required.

Most of the readers might answer “No, missing a radiographic diagnosis or have a complication during an interventional procedure is only an error. There is malpractice if there is no respect of the rules and/or standard protocols.”

However this answer may be not obvious for lawyers, judges, and patients who, although may be aware of the “human factor” and have understood the experts' opinion, often have not the right elements to determine if there happened an error or a malpractice in a specific situation. Caldwell and Seamone [14] addressed this issue claiming that the judges should focus on issues such as proof of competence (evidence that the radiologist has the right competencies in his daily practice), habits of practice (the radiologist showed safe practicing habits), and use of proper techniques (the radiologist respected the standard of care). On our advice lawyers and experts attending the process should leverage on those values which can give the answer if there has been malpractice or not.

## 3. How to Protect from a Malpractice Litigation?

Although in USA most of all litigated malpractice claims against radiologists are dismissed in court and only a low rate (3–5%; rates may vary by state [15]) go to verdict, long time to settlement and money which might be spent in a law process remain the main fears among radiologists [2, 16, 17]. Recently similar feelings seem to gain ground also in Italy and other European countries [18].

Those fears during a malpractice litigation may lead to the so-called “malpractice stress syndrome” which is reported to be quite common among sued radiologists as showed by US and Italian studies [19, 20].

In particular psychological reactions of malpractice syndrome are most frequently anxiety (63.8%) and anger (61.0%) [19]. But also feelings of helplessness (39.0%), disappointment (32.4%), distress (32.4%) humiliation (19.0%), and guilt (10.5%) have been reported [19].

Both US and Italian studies reported that clinical behavior of sued doctors may alter the professional conduct leading to “defensive medicine” practice thus increasing the cost of the whole health system [18–22].

For a diagnostic/interventional radiologist what are the antidotes to such problems?

The answer may be summarized in 4 points:

1. Follow as long as possible the standard of care.
2. Be careful in the off-label use of devices.
3. Improve communication skills with colleagues, nurses, technicians, and, overall, patients.
4. At last remember the insurance.

## 4. Standard of Care

Several radiological associations have disposed practice and quality improvement guidelines for any kind of imaging exam or interventional procedure.

Although the specific protocols of the standard practice are generally unknown by lawyers and judges, since 1905 the state of New York appeals court dealing with the general definition of the standard of care: *“The law requires a physician to possess the skill and learning which is possessed by the average member of the medical profession… and to apply that skill and learning with ordinary reasonable care. He is not liable for a mere error in judgment, provided he does what he thinks is best after a careful examination. He does not guarantee a good result”* [23].

However experts, choice is fundamental to give correct advices to the court, thus explaining the specific issues of the standard of care in that specific case.

Otherwise the court often reserves to itself the power to evaluate expert opinion in order to ensure that it is logical and defensible. 

In USA especially, patients tend to consider the standard of care as standard of perfection, that neither exists in medicine nor should be claimed by the radiologist or interventional radiologist before the radiologic exam.

All Radiology and Interventional Radiology societies as American College of Radiology (ACR), European Society of Radiology (ESR), Radiological Society of North America (RSNA), Cardiovascular and Interventional Radiological Society of Europe (CIRSE), and Society of Interventional Radiology (SIR) are strongly committed to address the medical-legal issues related to the radiologic profession.

In the interventional radiology field, defining standardized protocols of imaging exams and procedures is particularly needed. Thus both SIR and CIRSE have developed several practice guidelines to improve the standard of quality of numerous procedures [24, 25].

Apart from the specific issues regarding interventions, a correct and fluid workflow is necessary due to the well-known risk of error during the planning, execution of the procedure, and especially after the procedure when often a close management of the patient is paramount.

On this purpose National Patient Safety Agency joined with the Royal College of Radiologists and CIRSE have developed a Patient Safety Checklists for Radiological Interventions in order to standardize the workflow in the clinical practice, thereby reducing the risk of complications [26, 27]. Developing a patient safety plan they aim to reduce the errors related to the workflow and management of the patient.

ACR has recently endorsed the ACR Appropriateness Criteria [28] regarding both diagnostic exams and interventional procedures. We should note that those criteria are intended only “to guide radiologists, radiation oncologists and referring physicians in making decisions regarding radiologic imaging and treatment…. The ultimate decision regarding the appropriateness of any specific radiologic examination or treatment must be made by the referring physician and radiologist in light of all the circumstances presented in an individual examination.”

That statement means that generally the societies give the route to the physician who has to decide the behavior in a specific situation which is not always contemplated by the guidelines themselves; that is the clinical realty. Therefore the physician must respect the guidelines, and where the guidelines cannot help him, he should try to explain his decisions and actions as “prudent and reasonable.”

Finally training and updating are fundamental tools to fill the radiologist's standard of practice locker. 

In fact clinical competence has been defined as “…when a practitioner has sufficient knowledge and skills such that a procedure can be performed to obtain the intended outcomes without harm to the patient”; it constitutes an essential part of the standard of practice [29].

The radiologist should be retrained at regular intervals and continuously update his knowledge by getting training in new technologies. When the physician considers that he does not hold an adequate expertise, it is recommended not to perform a diagnostic or interventional procedure and to seek for help or refer the patient to another colleague.

## 5. Off-Label Use of Devices

In any hospital anywhere in the world many devices and drugs are daily used off-label [30, 31].

Interventional radiologists have invented and remodeled many new devices and technologies over the past years.

The off-label use is fine and valuable to the patient's benefit, as long as all goes in the correct way. Otherwise, if there is some trouble and the patient comes to harm, the subsequent legal action will be complicated by the off-label use itself. Herein only the expert witness can clarify whether the off-label use of a device was justifiable or not.

But what is the position of the main international societies of interventional radiology?

In 2007 SIR has stated its position as follows: “The Society of Interventional Radiology (SIR) confirms its strong support for the autonomous clinical decision-making authority of a physician. This includes the lawful use by a physician of an FDA approved medical device or drug product for an unlabeled indication when such use is based upon sound scientific evidence and/or sound medical opinion. The SIR affirms the position that, when the “off-label” use of a device or prescription of a drug represents safe and effective therapy, third party payers, including Medicare, should consider the intervention as reasonable and necessary medical care, irrespective of labeling, and should fulfill their obligation to their beneficiaries by covering such therapy, and be required to cover appropriate “off-label” uses of drugs on their formulary. …*omissis*” [32].

In the USA, FDA has a broad authority to regulate the marketing of drug, biologics, and medical devices. Nevertheless FDA has no authority to regulate the clinical practice of the physicians. Therefore the physicians are allowed to use FDA-approved or cleared products in any way they think adequate in the care of specific cases. On the contrary a physician is not allowed to engage in activities that would amount to marketing the off-label use. Such matter is addressed by the same FDA and several courts which have repeatedly recognized the propriety of the off-label use [31].

In Europe, the European Commission endorsed the Revised Directive 93/42/EEC concerning medical devices [33]. There is no malpractice if an off-label device has been used to fit a specific situation, whereas large scale off-label use or experimental use of devices without CE marking may be considered malpractice unless there is ethical committee approval and the patient has been thoroughly informed [34]. Recently in France has passed a law and a related decree entitled “Temporary Recommendations for Use” (TRUs; Decree no 2012-743, May 9, 2012) which has the advantage to encourage the development of possibly viable uses for marketed drugs and the monitoring of benefit-risk ratios for new indications [35]. This law should be potentially extended to the whole European Union.

## 6. Improve Communication Skills “...This Is Called Noncommunication...”

Clinical practice of radiology is characterized by continuous involvement in any kind of communication with patients and colleagues. However it may often happen, looking at a radiologic report or an exam request sheet from a referring physician, to read incomprehensible words, sentences, or abbreviations.

Moreover did not you ever come to know that a patient reported that he did not understand or have figured out different things about the radiological exam or interventional procedure that you have previously explained to him/her? Or is it ever happen that the patient is not satisfied how you explained the result of your radiologic exam or interventional procedure?

We might call those situations as “noncommunication” problems.

In a recent literature review of claims by Whang et al. from 1980 to 2010 [2], failure to communicate awarded the third position among the most frequent causes of malpractice lawsuit in radiology. They found a discrete frequency of lawsuits in case of failure of communication in different cases: failure to communicate properly with another provider accounted for 0.71 suits per 1000 person-years, and inadequate communication with the patient or the patient's family comprised 0.40 suits per 1000 person-years. Less frequent was failure to recommend further exams (0.41 suits per 1000 person-years).

Moreover in a UK report complaints expressing dissatisfaction with doctors' communication skills showed a steep increase in 2011 [36].

But what kind of communication to patients might get in trouble a radiologist?

The main types of talks are informed consent discussion, conveying bad/good news, and admitting medical error.

Informed consent actually represents a contract of duty of care between the patient and the radiologist. In 1997 International Convention on Human Rights and Biomedicine [37] in Chapter II from article 5 to 9 expressed the following rules regarding the informed consent.

Article 5. An intervention in the health field may only be carried out after the person concerned has given free and informed consent to it. This person shall beforehand be given appropriate information as to the purpose and nature of the intervention as well as on its consequences and risks. The person concerned may freely withdraw consent at any time.

Important articles regarding patients who cannot give their consent are the following.

Article 6.2. Where, according to law, a minor does not have the capacity to consent to an intervention, the intervention may only be carried out with the authorization of his or her representative or an authority or a person or body provided for by law.

Article 6.3. Where, according to law, an adult does not have the capacity to consent to an intervention because of a mental disability, a disease or for similar reasons, the intervention may only be carried out with the authorization of his or her representative or an authority or a person or body provided for by law. The individual concerned shall as far as possible take part in the authorization procedure.

Article 8: Emergency Situation. When because of an emergency situation the appropriate consent cannot be obtained, any medically necessary intervention may be carried out immediately for the benefit of the health of the individual concerned.

Those and the rest of international rules have been turned in proper state laws across different Countries for example, RCR endorsed a standard of practice document to provide guidance to radiologists involved in obtaining the informed consent [38].

But what is a legally adequate informed consent? Legal requirements of an informed consent may vary from state to state; however generally we can affirm that it is considered good practice to ensure that consent is given in the appropriate environment, in the proper manner, and in the presence of appropriate and relevant information [38].

Then to avoid malpractice the radiologist must be sure of the following.

(i) The patient has the right information to take a decision.
(ii) The information has been presented in a way/language that the patient can understand.
(iii) The patient has shared and is convinced of the process of the radiologic exam and agrees with its outcome.

On imparting bad news, often radiologists may focus on technical medical issues and place little emphasis on patient factors such as functional status, values, wishes, and fears [39]. Otherwise in case of good news, the situation seems to be more “easy” for the radiologist which should be at the same time optimist and balanced, remembering to recommend any future exams or possible developments of the current health status.

Contrary to ethical and legal expectations, physicians often are reluctant to disclose medical errors to their patients [40, 41]. 

Error disclosures consist in a delicate conversation that requires several communication skills, but physicians often lack the capacity to conduct these difficult debates. Communication skills are even more required in radiologists performing interventional/invasive procedures. In particular comprehensive information including therapy alternatives and potential complications is strongly recommended [42].

In Italy communication problems are considered in some criminal law proceedings (ex art. 589 or 590 of the penal code), which involve the radiologist and other specialists. Communication troubles have been found to account for more than 12% of causes of malpractice litigation against radiologists [3, 43].

Magnavita et al. [43] concerned about error disclosures conclude that it seems reasonable to explain and excuse about a mistake in order to reduce the patient's suffering and possibly the consequences of the damage caused. Unfortunately it seems that such behavior cannot reduce the risk to be sued by the harmed patient. 

Our advice to well face such situations is to fully explain complexity of the procedure/exam and its limitations and subsequently explain why and how the error happened.

In this way the error should become more “reasonable” for the patient or patient's family mitigating his/their expectations and demands.

However also integrating verbal with nonverbal communication is paramount as demonstrated by Hannawa [41]. The following behaviors belong to a proper non-verbal communication to facilitate positive error disclosure outcomes: nonverbal displays of immediacy (e.g., appropriate touching and physical distancing, direct body orientation, and prolonged gazes), expressiveness (i.e., appropriate physical and vocal animation), altercentrism (e.g., displays of attentiveness and interest in the patient, use of affirming head nods), and positive effect (e.g., appropriate smiling, vocal and facial pleasantness), and engagement in skillful nonverbal interaction management (e.g., allowing the patient to speak without interruptions).

Another matter is the communication with colleagues or health personnel: interpretation, reporting, and execution represent the most significant phases of the radiological medical act. Communication of radiologic reports/result of interventional procedures may be formal (routine communication, written) or nonroutine communication.

A correct formal report requires fundamental records competencies as proper knowledge, skills, and behavior which have been described by Wallis and McCoubrie [44].

They outlined, apart from the structure of the report, the importance of proofreading the report to avoid vague reports and typing or voice recognition errors which may lead to a malpractice litigation.

ACR guideline [45] defines the standard of report: “the final report is the definitive documentation of the results of an imaging examination or procedure.”

Regardless of the manner how the report is written and transmitted it is fundamental that the report should be transmitted in time in order to allow the right care by the referring physician, and also they agree that “the final report should be proofread to minimize typographical errors, accidentally deleted words, and confusing or conflicting statements and use of abbreviations.”

In case of emergency (i.e., massive pulmonary embolism) the radiologist has to provisionally report his diagnosis to allow timely therapy by the referring clinician as recommended by the ACR guidelines about radiologic communication [45]. Despite this kind of communication is informal is advisable to document it [27, 28].

But how to document it? Communication by telephone or in person to the treating or referring physician or his/her representative is appropriate and assures receipt of the findings. But as the famous dictum says “verba volant… scripta manent,” is advisable to place documentation of the non-routine communication in the subsequent formal radiology report or in the patient's medical record or may be entered in a department log and/or personal journal [11, 45]. 

Medical confidentiality is a very common issue related to the communication in radiology.

Generally, confidentiality is defined as someone consciously and voluntarily revealing to a confident potentially harmful information, in the understanding that this shall not be further disclosed without the confider's explicit consent [46]. Medical confidentiality also involves involuntary information transfer between physician and patient. Radiologists as other physicians have ethical and moral responsibility to respect patient's right to confidentiality.

Breach in confidentiality may be rarely due to intentional disclosure of patient information or more frequently to unauthorized disclosure to third parties and discussion in public areas of the hospital.

Moreover disclosure of clinical information with research or teaching purpose, during meetings and continuing education courses, may lead to patient's identification. In this cases patient's permission is needed, otherwise images should be anonymized [47].

Due to the recent development of digital imaging techniques, images are promptly available and storable on computers. Also the data transfer is made easier by using telecommunication networks. However, this advancement rises important problems about data security.

Privacy is ensured using data signature, authentication, encryption, and certificate exchange; nevertheless further control measures are needed to enforce confidentiality [48].

Finally all aspects of communication may be considered the pillars for the survival of the radiologist, so if those pillars are not so robust, he may often fall in a lawsuit.

## 7. Liability Insurance

Several insurance companies and brokers exist worldwide with different malpractice policies. However generally when a radiologist signs a contract of professional liability insurance, he should read the contract in every part, being aware of the losses covered by the agreement and those that are excluded. Nevertheless it is fundamental that radiologists have a reasonable understanding of the extent and limitations of the professional liability contract before signing it.

But how do generally the insurances work? What they usually cover for radiologists?

In many states, most of the malpractice insurance is provided through companies owned by the medical association or other physician groups (i.e., SIRM is the Italian Society of Medical Radiology which is a carrier of different insurance brokers) [51].

Generally two basic types of professional liability policies are available: “occurrence” and “claims made.”

Professional liability insurance policy covers only claims of alleged malpractice reported during the policy period, regardless of when the service was rendered. Otherwise “occurrence” basis means that policy covers which claims of alleged malpractice occurred during the policy period, regardless of when the lawsuit was filed. 

However an insurance based on “claims made” covers the physician only if the incident and the filing of the claim happen while the policy is in effect.

If any suit is filed later, you drop the claims-made policy, so you are not covered for unless you pay for what is known as “tail coverage,” the term used for an extended reporting endorsement. Note that tail coverage is often expensive (i.e., three times the annual premium) [52]. Otherwise “occurrence” policy can cover for a lawsuit filed after the policy time is over, if the incident happened in past during the period of covering.

We should note the in most of the cases insurance contract neither covers the criminal prosecution nor a wide range of potential liabilities under civil law that are not enumerated in the policy, but which may be subject to other forms of insurance.

So the radiologist should consider the following two issues before the insurance choice.

1. Which malpractice insurance carrier should the radiologist purchase coverage from.
2. The amounts of malpractice coverage.

There are important elements to check in an insurance carrier policy:

(i) coverage of defense costs (they include fees of the defense attorney retained by the insurance company, the fees of expert witnesses, court reporters' fees, and clerical expenses).
(ii) premium costs, the company's fiscal soundness, claims handling, and sensitivity to policy holders.

Useful sources to find an insurance company that suits your practice are the following.

### 7.1. In the USA

Physician Insurers Association of America, a trade association of more than 50 professional liability insurance companies owned and operated by doctors and dentists: http://www.piaa.us/.

### 7.2. Worldwide

AM Best Company, an independent industry analyst with carrier ratings that are considered an industry benchmark: http://www.ambest.com/.

### 7.3. In the EU

The Organisation for Economic Cooperation and Development (OECD): http://www.oecd.org/.

Regarding the malpractice coverage amount, it should reflect and match your practice.

Generally the insurance carriers set up the premium and the coverage amount in relation to the medical specialty, geographic location, and claims history.

Radiology is not one of the highest risk medical specialties for malpractice litigation, but it is certainly not the lowest [53, 54]. Radiologists are frequently involved in malpractice lawsuits in which it is not always easy to understand exactly how the radiologist became involved [4].

Moreover subspecialties as interventional radiology and gastrointestinal radiology have additional risks, as compared to the classical diagnostic radiology, coming from the deeper interaction with patients. Thus for such sub-specialties a higher coverage and related premium are advised. However companies often do not differentiate those sub-specialties from others including the whole radiology specialty into one risk level.

Finally good practices concerning the liability insurance are the following.

(i) Read and complete your insurance application thoroughly and accurately.
(ii) Always keep a copy of your insurance coverage as a proof that you are insured!
(iii) Verify all terms of coverage/exclusion of your policy.

## 8. Conclusion

Radiology is nowadays a branch of medicine increasingly involved in the clinical management of the patient and thereby in the “duty of care.” Awareness of the main medical-legal issues and concepts (see Table 1 for useful medical-legal terms and concepts) in the radiology field may affect the behavior of both radiologists and referring physicians to improve the global care of the patient reducing the risk of errors and troubles related to malpractice litigation.

## Figures and Tables

**Table 1:** Useful medical-legal terms and concepts.

Term	Definition
Abandonment [49]	Legally indicates both the intention to abandon and the external act by which the intention is carried into effect. In medicine means termination of a physician-patient relationship without reasonable and without an opportunity for the patient to acquire adequate medical care, resulting in some type of damage to the patient.

Answer [49]	A legal document with a defendant's written response to a complaint or declaration in legal proceedings.

Affidavit [49]	Voluntary, written statement of facts made under oath before an officer of the court or before a notary public.

Battery [49]	In medical malpractice it is a contact of some type with a patient who has not consented to the contact. Battery may be considered either a civil or a criminal offense.

Complaint [49]	A legal document that is the initial pleading by the plaintiff in a civil action.

Common law [49]	The body of law passed down to the American colonies by the British legal system and has been interpreted and refined by case law.

Duty [49]	An obligation recognized by the law. A physician's duty to a patient is to provide the degree of care ordinarily exercised by physicians practicing in the same community or area of specialization.

Expert witness [29]	Is a witness who has expertise and specialised knowledge in a particular subject; it is sufficient that others may officially and legally rely upon the witness's specialized opinion about an evidence or fact occurred.

Hedging [44]	Is an ambiguous statement which may lead to write a vague report.

Insurance broker	Brokers and agents are the retailers of the insurance. They assist insureds in developing risk management strategies appropriate for their risk profile.

Licensing [44]	A state specialty board grants a license to an individual physician, which gives that physician the right to practice in his or her specialty field.

Patient autonomy [38]	A doctor should find out what patients want to know and ought to know and respect the patient's decision.

Professional competency [50]	Habitual and judicious use of communication, knowledge, technical skills, clinical reasoning, emotions, values, and reflection in daily practice for the benefit of the individual and community being served.

Prudent doctor [38]	The doctor weighs the risk of a certain complication occurring against the risk resulting from putting a patient off necessary treatment.

Prudent patient [38]	Concept recently developed in the USA that focuses on what the average “prudent patient” would want to know about potential risks and treatment options.

Res ipsa loquitur [49]	“The thing speaks for itself.” A case in which the personal injuries or property damage would not have occurred without negligence. This theory allows a patient to prove his or her case without the need of an expert witness to testify that the defendant violated the standard of care.

Timely [49]	Promptly; within a reasonable period of time, with “reasonable” being judged in terms of the particular circumstances of a case.

Tort law [49]	A body of rights, obligations, and remedies that is applied by courts in civil proceedings to provide relief for persons who have suffered harm from the wrongful acts of others.
